# Precautions in Dispensing Poisons, Etc.

**Published:** 1881-05

**Authors:** Robert F. Fairthorne


### PRECAUTIONS IN DISPENSING POISONS, ETC.

BY ROBERT F. FAIRTHORNE, PH G.

The caution necessary in dispensing poisons can scarcely be too great, not only on account of the protection which the public expects of us as pharmacists, but on account, also, of that which is due to our own interests. When we take into consideration the fact that a fellow mortal may be deprived of life, or rendered an invalid for years, through some dangerous article being taken by mistake for some innocent substance, through not being marked or labeled, it becomes us to consider well the best means for insuring safety. Every safeguard should be employed.

When dry articles of a poisonous character are dispensed, not only should they be labeled, but wrapped up in at least two papers, *both* of which should have printed labels of the name of the article on it, and the word poison in bold letters. In addition to this the inner wrapper should have the name of the article and the word poison *written* on it. By this plan, even if the labels should be lost or removed, there would still be a means for identifying the drug, and life, perhaps, saved.

This, too, might prove to be of value as evidence in poisoning cases, and it might be well if such a custom was universally adopted.

In dispensing prescriptions containing poisonous articles it will not always be advisable for the pharmacist to mark poison on the bottle; a label with the word caution on it can, however, and should always be pasted on the bottle or package. The physician frequently orders such articles as arsenic, aconite and corrosive sublimate, in appropriate doses, and if the mixtures containing them were labeled poison many nervous people would not take the medicine ordered for them. The practice of prescribing such substances as these in a concentrated form such as tincture of aconite, Fowler's solution, solution of the iodides of arsenic and mercury, has been frequently followed by fatal doses, such as a teaspoonful or more being taken by the patient in mistake for other liquids.

It has been proposed by some persons, unacquainted with either the pharmaceutical or medical profession, that all bottles and drawers should be labelled in English, as a means for preventing mistakes. I will venture to assert that no one with long experience in either profession can be found who believes that this would be the slightest safeguard; on the contrary, I believe that the danger of mistakes being made would be increased thereby, as there is greater similarity of terms, or in sounds of names, in English than in Latin. Black drop and black draught, for instance, ladanum and laudanum; chamomile, calomel, calamine, calamus; Jamestown weed and gentian, the former being frequently pronounced jimsen, can be named as having some similarity; May weed and May apple, antimonious tartrate of potassa and acid tartrate of potassa, and many other articles. Not only would there be danger from this cause, but from another, namely, the overconfidence in their own capabilities and discretion, which leads some individuals without adequate experience and knowledge to assume responsibilities not belonging to them, from which cause more mistakes have been made than from any other. No one whose mental faculties are so small as not to be able to become as familiar with the Latin used in naming drugs as though written in English should undertake to study the chemical and therapeutical characteristics and doses of medicines used with the view of prescribing or dispensing the same, as such intellects would not be capable of retaining sufficient information to enable them to do so with propriety.

The practice of dispensing medicines of a poisonous character in peculiar bottles, such as the corrugated, sanded and stellated styles, or bottles covered with diamond-shaped points, is to a certain extent a good one, if generally adopted; but until some law is passed compelling their use I think their employment may be open to objection on account of some druggists using such bottles and others not using those so marked, and because customers who had become accustomed to the use of these peculiar bottles for poisons only might fall into the error of considering all medicines dispensed in ordinary bottles as non-poisonous.

The necessity of a knowledge of the chemical reactions of poisons in compounding prescriptions is apparent. The neglect to paste the label "shake the vial" on the bottle containing strychnia has been the cause of death through the patient taking an overdose when the last dose in the bottle was given, as nearly all the strychnia in the prescription was precipitated. This precipitation would take place in the presence of many substances in common use when solutions of strychnia, atropia, morphia, etc., are mixed with them, as Huxham's tincture, salicylate of sodium, etc. Again, some innocent substances are converted into a dangerous one, as calomel into a corrosive poison, when mixed with chloride of ammonium, nitro-muriatic or hydrocyanic acids.

Where there are two substances bearing considerable resemblance to each other, such as morphia sulphate and quinia sulphate, it is perhaps as well to keep them in bottles that differ both in appearance and size. It has been my practice to dispense quinia from the bottle as it comes from the manufacturer, whilst the morphia has been kept in a blackened bottle. It is also a good plan to paint the store bottles containing powerful poisons black, as a distinguishing mark, and those that are very strong, such as aconitia, atropia, etc., if kept in small vials and these again placed inside larger bottles can scarcely be dispensed in mistake for other substances.

Contrary to the practice in many stores, I believe that mistakes can be avoided in many cases, or, I may say in most cases, by leaving the bottles from which medicines have been taken for use in prescriptions on some part of the counter, especially devoted for the purpose, so that they may be examined before the customer leaves the store, and each bottle compared with the ingredients of the prescription. This practice, although accompanied with some inconvenience, enables the proprietor or superintendent of the store to inspect each article employed in compounding so that nothing need be left to the responsibility of inexperienced persons, whereas this cannot be done if the bottles are taken from the shelf and instantly replaced, unless some one one could follow each movement of each individual so employed.

Another practice, which I think a good one, is to paste poison or caution labels on both sides of the bottles that required to be so marked when sent out of the store, so that there will be an additional safeguard for the customer, and in this connection I would state that I think the practice of selling laudanum without being marked poison is very reprehensible.

In addition to these precautions, I think the following articles, which are in extensive use, when sold should be labeled with the word caution, namely: spirits of hartshorn, acetic acid, aromatic sulphuric and tartaric acid, chloroform, ether and benzin (on account of inflammability), bitter almonds, bitter almond oil (water and extract), tartar emetic and its preparations, cherry laurel water, weak solutions of carbolic acid (strong should be marked poison), wine of colchicum root, strong solutions of chloride of zinc, tinctures of digitalis and conium, tincture of iodine, santonin and the preparations containing it, artificial fruit extracts, alcohol, Goulard's extract, podophyllin, and as a general rule all liniments not containing poison; the latter, of course, should be so labeled.

I have written thus at length, feeling that one of the most important subjects and one requiring very great attention is, care in dispensing poisons.

